# Retraction: Berberine inhibits invasion and metastasis of colorectal cancer cells via COX-2/PGE_2_ mediated JAK2/STAT3 signaling pathway

**DOI:** 10.1371/journal.pone.0328009

**Published:** 2025-07-14

**Authors:** The PLOS One Editors

After this article [1] was published, concerns were raised regarding Figs 2, 3, 5 and 6.

Specifically:

- There appears to be partial or full overlap between the following panels:
  - The Migration - sh-1/COX-2 panel in the LoVo cells of Fig 2E and the Invasion - AZD1480– panel in the LoVo cells of Fig 3C.
  - The p-JAK2 panel in LoVo cells of Fig 3A in [1] and the CD44 panel in Fig 4 (e) of [2] (Retracted in [3]).
  - Lanes 1-4 of the Stat3 panel in LoVo cells of Fig 5C and lanes 1-4 of the β-actin panel in LoVo cells of Fig 5D, when flipped horizontally and with altered aspect ratio.
  - The Stat3 – Vector panel in the SW620 cells of Fig 6A in [1] and the β-actin panel in Fig 6 (g) of [2] (Retracted in [3]).
- There appear to be vertical discontinuities in the following panels of [1]:
  - Between lanes 1 and 2 of the p-Stat3 panel in SW620 cells of Fig 5A.
  - Between lanes 1 and 2 of the p-Stat3 panel in LoVo cells of Fig 5A.

Regarding the overlap between Figs 2E and 3C, the first author stated that the Migration - sh-1/COX-2 panel in the LoVo cells of Fig 2E is correct, but was mistakenly duplicated in Fig 3C and therefore the Invasion - AZD1480– panel in the LoVo cells of Fig 3C is incorrect. The first author provided underlying images for Fig 2E, however, PLOS identified what appears to be partial and full panel overlap within this set of data, with the shRNA/NT panel in Fig 5C of [4], and with panels in Figs 2C, 4B and 6A of [5].

The first author also provided underlying images and individual-level quantitative data for all other figures in [1] and replicate data completed at an unknown date for Fig 3C, for which PLOS also identified what appears to be partial panel overlap within panels of this dataset.

Given the nature and extent of the above unresolved concerns which bring the reliability and integrity of the results and conclusions into question, the *PLOS One* Editors retract articles [1] and [5]. The retraction notice for [5] can be found in [6].

PLOS also noted that the methods section of [1] does not appear to report the method of euthanasia, humane endpoints, or if any control animals died during the study.

All authors either did not respond directly or could not be reached.
